# Impact of hypertension on somatic pain sensitivity in chronic headache

**DOI:** 10.1186/1129-2377-14-S1-P160

**Published:** 2013-02-21

**Authors:** ET Tafuri, S Di Fabio, E Cozza, GP Affaitati, A Fabrizio, C Tana, L Felicioni, M Bucci, A Mezzetti, MA Giamberardino

**Affiliations:** 1 Headache Center and Center for the Study of Hypertension, G. D’Annunzio University of Chieti, Italy; 2 Headache Center, G. D’Annunzio University of Chieti, Italy; 3 Center for the Study of Hypertension, G. D’Annunzio University of Chieti, Italy

## Aim of investigation

Previous studies have shown that chronic headache (migraine and/or tension-type; CH) is characterized by diffuse somatic hyperalgesia, while arterial hypertension (HY) produces somatic hypoalgesia which is only partly attenuated by antihypertensive treatment. The aim of the study was to assess if comorbidity between CH and HY results into an attenuation of the hyperalgesia due to headache.

## Methods

Forty-eight patients of both sexes [28-56 years] with moderate essential hypertension plus chronic headache (HY+CH) were examined. Twenty-two received no treatment for hypertension, the remaining 26 were under therapy, with good control of pressure values. All underwent measurement of a) blood pressure at rest; b) pressure and electrical pain thresholds in the trapezius, deltoid and quadriceps muscle of one side. The results in this group were compared with those of 40 healthy control subjects (C), 52 patients with chronic headache without hypertension (CH) and 190 patients with hypertension without headache (82 without and 108 with treatment)(HY)(all age and sex-matched).

## Results

Immediately before threshold evaluation, untreated hypertensive patients had higher than normal blood pressure levels, while hypertensive patients under treatment, headache patients without hypertension and control subjects had normal blood pressure values. Pain thresholds at all sites in HY+CH were significantly lower than normal and HY in both treated and untreated patients (p<0.001). They were not significantly different from thresholds recorded in CH.

## Conclusion

Comorbidity between chronic headache and arterial hypertension (with or without antihypertensive treatment) does not involve any attenutation of the typical diffuse hyperalgesia that characterizes chronic headache. These results suggest that the sensitization process behind diffuse hyperalgesia in chronic pain forms such as chronic headache prevails on the hypoalgesia-determining mechanisms of hypertension in comorbid patients.
